# Motoric Cognitive Risk Syndrome Using Three-Item Recall Test and Its Associations with Fall-Related Outcomes: The Korean Frailty and Aging Cohort Study

**DOI:** 10.3390/ijerph17103364

**Published:** 2020-05-12

**Authors:** Hayoung Shim, Miji Kim, Chang Won Won

**Affiliations:** 1 Department of Biomedical Science and Technology, Graduate School, Kyung Hee University, Seoul 02447, Korea; hayoung0517@khu.ac.kr; 2 Department of Biomedical Science and Technology, College of Medicine, East-West Medical Research Institute, Kyung Hee University, Seoul 02447, Korea; 3 Elderly Frailty Research Center, Department of Family Medicine, College of Medicine, Kyung Hee University, Seoul 02447, Korea

**Keywords:** motoric cognitive risk syndrome, fall, gait speed, cognitive function, three-item recall, older adults

## Abstract

Motoric cognitive risk (MCR) syndrome is originally defined as the presence of subjective cognitive complaints (SCCs) and slow gait (SG). MCR is well known to be useful for predicting adverse health outcomes, including falls and dementia. However, around four out of five older Korean adults reported SCCs, thereby, it may not be discriminative to define MCR in Korea. We adopted the three-item recall (3IR) test, instead of SCCs, to define MCR. This cross-sectional analysis included 2133 community-dwelling older adults aged 70–84 years, without dementia or any dependence in activities of daily living from the Korean Frailty and Aging Cohort Study. The newly attempted criteria of MCR using 3IR were met by 105 participants (4.9%). MCR using 3IR showed synergistic effects on fall-related outcomes, whereas the conventional definition of MCR using SCCs was not superior to SG only. MCR using 3IR was associated with falls (odds ratio [OR]: 1.92; 95% confidence interval (CI): 1.16–3.16), recurrent falls (OR: 2.19; 95% CI: 1.12–4.32), falls with injury (OR: 1.98; 95% CI: 1.22–3.22), falls with fracture (OR: 2.51; 95% CI: 1.09–5.79), fear of falling (OR: 3.00; 95% CI: 1.83–4.92), and low activities-specific balance confidence (OR: 3.13; 95% CI: 1.57–6.25). We found that MCR using 3IR could be useful in predicting fall-related outcomes in a cultural background reporting more SCCs, such as Korea.

## 1. Introduction

Substantial links have been reported between cognition and gait, and the combination of these two factors has been conceptualized by motoric cognitive risk (MCR) syndrome [1,2]. Compared to each component alone, MCR has stronger predictive validity for adverse health outcomes, such as dementia, falls, disability, and death [3,4,5].

MCR was originally defined as the presence of subjective cognitive complaints (SCCs) and slow gait speed, without dementia and any dependence in activities of daily living [3]. Of these criteria, most studies have defined slow gait as below one standard deviation of the usual gait speed established in each cohort according to age and sex [6]. To the contrary, many previous studies flexibly adapted various criterion of SCCs to suit their research environments, i.e., from standardized questionnaires regarding general cognitive performance, from one simple self-rating question regarding memory function, from cut-off scores used in several objective cognitive function tests, or from informant reports on subjects’ cognitive problems [5,6]. The various criteria of SCCs in previous studies might have influenced the inconsistent results [6]. In particular, there were different results on the association between MCR and subtypes of dementia. Verghese et al., reported that MCR was strongly associated with vascular dementia (VaD), but not with Alzheimer’s disease (AD), when identifying SCCs using comprehensive objective assessments [3]. By contrast, in some studies using self-rating questions on memory [7,8], MCR was associated with increasedrisk of AD [9].

MCR is expected to be useful for various settings because the assessments of each component, presence of SCCs and gait speed, are relatively convenient [9]. However, several studies have reported limitations of SCCs that may be influenced to a greater extent by depression, personality, or cultural differences, than actual cognitive performance [10,11,12]. Similarly, MCR, which includes SCCs among the criteria, was associated with anxio-depressive disorders, depression [13] and personality, particularly neuroticism [14]. Furthermore, a number of studies have reported disparities between SCCs and objective cognitive function, with robust evidence that depressive symptoms seemed to have the greatest influence on SCCs [12].

Moreover, several studies reported that older Korean adults may have a cultural tendency to report SCCs more frequently [15,16]. The prevalence of SCCs in community-dwelling older adults in Korea was 77.7% [16]. SCCs may not be discriminative in Korea. The tendency of high reporting of SCC in older Korean adults may stem from culturally based factors, such as high prevalence of depressive symptoms [15,17,18].

MCR was found to predict adverse health outcomes [4]. Particularly, MCR is well known to be associated with falls and their recurrence, or post-fall fractures [19,20,21]. Therefore, we aimed to explore another criterion for the cognitive aspects of MCR, based on fall-related outcomes. As most previous studies have identified SCCs in memory [22], we introduced three-item recall (3IR) test, instead of SCCs, for defining MCR. 3IR test is a simple and well validated objective memory test. We investigated the associations between MCR and comprehensive fall-related outcomes by comparing the new MCR using 3IR, and original MCR using SCCs.

## 2. Materials and Methods 

### 2.1. Study Population

**Figure 1:**
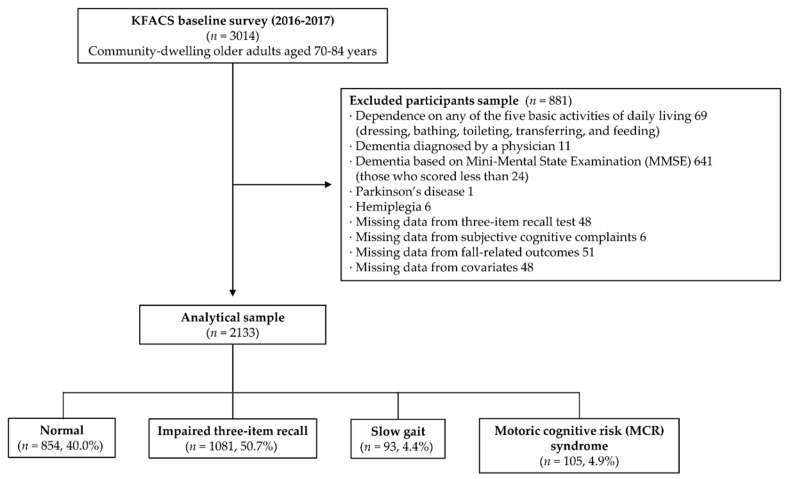
Flow chart of the study population.

The Korean Frailty and Aging Cohort Study (KFACS) is an ongoing prospective cohort study to investigate the frailty status of older Korean adults [23]. The KFACS recruited 3014 community-dwelling older adults aged 70–84 years from 10 nationwide centers, including rural, suburban, and urban areas, in a 2-year comprehensive baseline survey. Among the participants of the KFACS, those who were dependent in any of the basic activities of daily living (ADL) (*n* = 69), self-reported a diagnosis of dementia (*n* = 11), or scored < 24 points in the Mini-Mental State Examination (MMSE; *n* = 641) [24], and had a history of Parkinson’s disease (*n* = 1) or hemiplegia (*n* = 6), were excluded from the present study. In addition, those who had missing 3IR test scores (*n* = 48), a question about SCCs (*n* = 6), and fall-related outcomes (*n* = 51) and covariates (*n* = 48), were excluded. On the result, a total of 2133 older adults were selected for the present study (Figure 1). The Clinical Research Ethics Committee of Kyung Hee University Hospital approved the KFACS protocol (Institutional Review Board [IRB] number: 2015-12-103). The present study was exempt from the requirement for IRB approval by the Clinical Research Ethics Committee of the Kyung Hee University Medical Center (IRB No.: 2020–03–073).

### 2.2. Definitions of Motoric Cognitive Risk (MCR) Syndrome

#### 2.2.1. Original MCR using Subjective Cognitive Complaints (SCCs)

A single question extracted from the Korean Version of Short Form Geriatric Depression Scale (SGDS-K) was used to ascertain SCCs: “Do you feel you have more problems with memory than most?” [25,26]. A positive response, “yes”, to this question was defined as indicating the presence of SCCs [4,19,27]. Slow gait was defined as one standard deviation (SD) or below the age- and sex-specific mean values established in the KFACS [16]. The participants were asked to walk a 7 m distance, with initial acceleration and terminal deceleration sections of 1.5 m in their normal pace, and gait speed was calculated at the speed (m/s) of walking 4 m, the middle section of 7 m, using an automatic machine (Gaitspeedometer, Dyphi, Daejeon, Korea).

#### 2.2.2. New MCR using three-item recall (3IR)

The 3IR test of MMSE was administered to identify the deficits in memory function [28]. The examiner named three unrelated objects clearly and slowly, then asked the participant to name all three of them. The examiner repeated the words up to three times until the participant learned all of them, if possible. A few minutes later, the participant was asked to recall the three words as much as possible, without any hint. Scores were calculated from 0 (incorrect) to 1 (correct) for each item, where higher score indicates better ability. The sum of the 3IR tests was used to determine cognitive aspects of MCR. A score < 3 was considered to indicate deficits in memory recall [29]. Slow gait (SG) was still defined as one or more standard deviation (SDs) or below the age- and sex-specific mean values established in the KFACS [16].

### 2.3. Definitions of Fall-Related Outcomes

We collected the following six types of fall-related information using a standardized questionnaire: experience of a fall in the past 1 year, recurrent falls, falls with injury, falls with fracture, fear of falling, and low activities-specific balance confidence.

Experience of falls, recurrent falls, and falls with injury or fracture during the past 1 year were investigated. A fall was defined as an event that resulted in a person coming to rest unintentionally on the ground, not as a result of a major intrinsic event (such as stroke or syncope) or overwhelming hazard [30]. In addition, falls caused by acute medical events, such as sudden onset of paralysis and epileptic seizure, or excess alcohol intake, were excluded [31]. Recurrent falls were defined as two or more falls in the past 12 months. Falls with injury were defined as those where the participants reported sprains, bruises, lacerations, and fractures after falls. When a participant reported fracture as a consequence of a fall, it was defined as fall with fracture.

Fear of falling (FoF) was assessed using a question presented with five response choices: “Are you usually afraid that you may fall?” If participants answered “considerably” or “very much,” they were considered to have a FoF. Participants who responded “not at all,” “a little,” or “don’t know” were considered to have no FoF [32]. 

Activities-specific balance confidence (ABC) was administered using a 16-item ABC scale in which participants rated their balance confidence when doing specific activities [33]. Scores ranged from 0 (no confidence) to 100 (complete confidence). A higher score indicated greater confidence, and the total score was calculated as the average of 16 items. Low ABC was defined as ABC scale score ≤ 58.13 [34].

### 2.4. Measurements

All participants were interviewed based on standardized surveys for collecting information, and were examined using health assessments. The trained investigators obtained sociodemographic and lifestyle information: education level, type of residence, living conditions, marital status and whether they receive social security aid, smoking status, alcohol intake, and physical activity level. Low physical activity was defined as < 494.64 kcal/week for men and < 283.50 kcal/week for women, using the International Physical Activity Questionnaire (IPAQ), values of which correspond to the lowest quintile (20%) of the total consumed energy established in a general population study of Korean older adults [35]. We inquired about the general health and medical history of each participant: body mass index (BMI), number of drugs taken daily, and medical conditions. Diseases were self-reported diagnoses by a physician, and comorbidities were defined as two or more of the following diseases: hypertension, diabetes mellitus, dyslipidemia, myocardial infarction, congestive heart failure, angina pectoris, cerebrovascular disease, peripheral vascular disease, osteoarthritis, rheumatoid arthritis, osteoporosis, asthma, and chronic obstructive pulmonary disease. Visual impairment was determined when the maximum value of left and right vision was < 0.3 [36]. Hearing impairment was identified as the minimum of the average value of left and right hearing exceeding 40 dB [37]. Instrumental activities of daily living (IADL) disability was determined when participants did not answer “completely independent” for one or more of the 10 activities using the Korea Instrumental Activities of Daily Living Scale (K-IADL) [38]. Nutritional status was determined using the Korean version of the short-from Mini-Nutritional Assessment (MNA-SF) [39]. Global cognitive function was assessed using the MMSE in the Korean version of the Consortium to Establish a Registry for Alzheimer’s Disease Assessment Packet (MMSE-KC) [40]. Depressive symptoms were assessed using the SGDS-K [41]. Participants were asked whether they perceived their health status as poor, fair, good, very good or excellent; “poor” or “fair” responses to the question were defined as fair/poor self-perceived health. Quality of life was evaluated using EuroQol five-demension scale (EQ-5D) [42]. Participants took the following physical function tests: handgrip strength, usual-pace gait speed, timed up and go (TUG) test [43], and short physical performance battery (SPPB) [44].

### 2.5. Statistical Analyses

Descriptive statistic analyses were conducted to compare the participants’ characteristics according to MCR status. The Chi-square test or Fisher’s exact tests was used for categorical variables, with adjusted standardized residuals as appropriate. In addition, after employing Levene’s test to determine homogeneity of variances, one-way analysis of variance (ANOVA) with Bonferroni post hoc test, or Welch’s ANOVA with Games–Howell post hoc tests, for continuous variables were conducted. Participants were divided into four groups for each definition: (1) MCR using SCCs: normal, SCCs only, SG only, and MCR using SCCs; (2) MCR using 3IR: normal, impaired 3IR only, SCCs only, SG only, MCR using 3IR. Multiple logistic regression analyses were performed to investigate the associations between MCR status and fall-related factors. We adjusted confounding factors by dividing the models into four steps. First, we adjusted for the recruited center and sociodemographic factors in Model 1: the recruited center, age, sex, low educational level, residence area, living alone, without partner, and social security aid recipient. Second, we further adjusted for lifestyle-related factors in Model 2: current smoker, alcohol consumption (≥ 2 to 3 times/week) and low physical activity level. Third, further adjustments were conducted regarding general health and medical conditions in Model 3: BMI, number of drugs taken daily, number of diseases, urinary incontinence, visual impairment, hearing impairment, poor nutritional status, and IADL disability. In the final model, Model 4 adjusted for the same factors as Model 3, with the addition of psychological factors: depressive symptoms and fair/poor self-reported health. All analyses were performed using SPSS (ver. 25.0; IBM Corp., Armonk, NY, USA). In all analyses, two-sided *p* < 0.05 was taken to indicate statistical significance.

## 3. Results

### 3.1. Descriptive Characteristics of the Study Population

Characteristics of participants according to new MCR status using 3IR are shown in Table 1. Sociodemographic factors including sex, education level, residence area, living alone, without partner, and status of receiving social securities, were significantly different between groups (*p* < 0.05). However, the age of the participants was not significantly different between the groups. Participants with MCR using 3IR showed significantly lower physical activity level than the normal group (*p* < 0.001). Moreover, compared to the normal group, the MCR using 3IR group was taking more medicines per day, and had more diseases, especially diabetes (all *p* < 0.01). The disabilities on any of the IADL were significantly different among the groups (*p* = 0.01). With regard to psychological factors, the MMSE score was the highest in the normal group and the lowest in the MCR using 3IR group (*p* < 0.001). Individuals with SG only or MCR using 3IR were more likely to rate their health status as poor and to have depressive symptoms than other groups (all *p* < 0.001). In all physical function tests, including handgrip strength, usual gait speed, TUG and SPPB, new MCR using 3IR showed poorer performance compared to the normal or impaired 3IR group (*p* < 0.001). Meanwhile, characteristics of participants according to original MCR status using SCCs are shown in Table S1.

**Figure 2:**
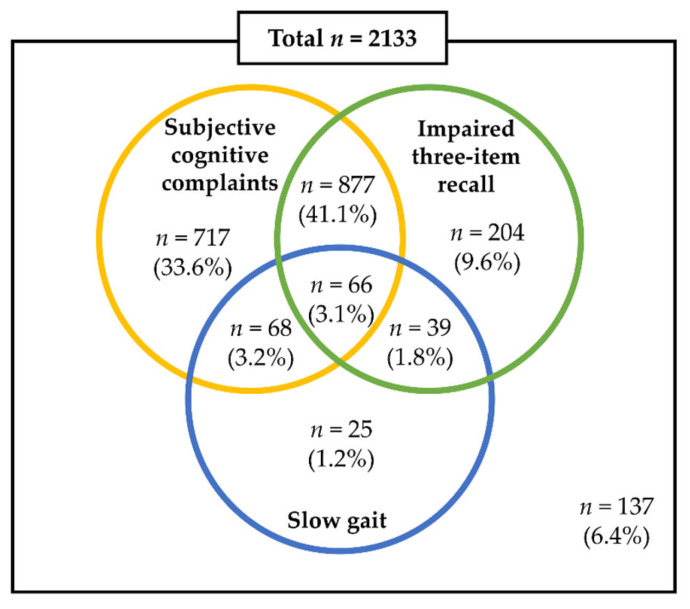
Venn diagram for the criteria and their overlaps.

Of the total of 2133 older adults in this cohort study, 105 (4.9%) met the newly developed MCR criteria using 3IR, of whom 55.6% had impaired 3IR and 9.2% had SG (Figure 2). Of the participants, 134 (6.3%) had MCR defined conventionally using SCCs, and SCCs were present in 81.0% of the all participants. A total of 943 participants (44.2%) had both impaired 3IR and SCCs.

**Figure 3:**
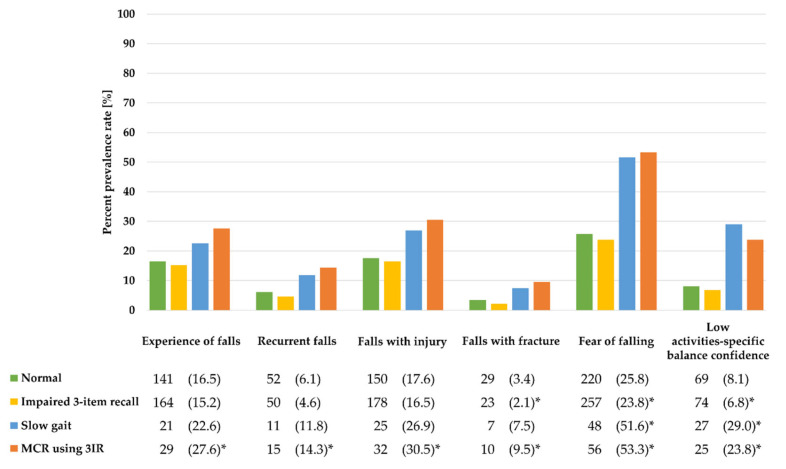
The prevalence of fall-related outcomes according to MCR status using 3IR. The numbers and percentages of outcomes are presented as *n* (%). * *p* < 0.0063.

The prevalence of fall-related outcomes according to MCR status using 3IR are presented in Figure 3. The MCR group using 3IR showed the highest prevalence of falls, recurrent falls, and falls with injury among the groups (all *p* < 0.0063). The prevalence of falls with fracture was lowest in the impaired 3IR only group, and highest in the MCR using 3IR group (all *p* < 0.0063). FoF and low ABC were significantly different among the groups (all *p* < 0.0063), with higher prevalence in the SG only or MCR using 3IR group.

### 3.2. Associations of MCR using 3IR or MCR using SCCs with Fall-Related Outcomes

Multiple logistic regression analyses were performed to investigate the associations between MCR and fall-related outcomes. Those for MCR using 3IR are shown in Table 2, and those of MCR using SCCs are presented in Table 3. Compared with the individual component of MCR using 3IR alone (i.e., impaired 3IR only and SG only), MCR using 3IR was only significantly associated with experience of falls in the past 1 year (OR: 1.92, 95% CI: 1.16–3.16), recurrent falls (OR: 2.19, 95% CI: 1.12–4.32), falls with injury (OR: 1.98, 95% CI: 1.22–3.22) and falls with fracture (OR: 2.51, 95% CI: 1.09–5.79) after considering all confounding factors. Moreover, the group with SG only (OR: 2.22, 95% CI: 1.31–3.75) and MCR using 3IR (OR: 3.00, 95% CI: 1.83–4.92) showed a significant association with FoF after adjusting for all confounders. With regard to low ABC, a significant association was observed in the groups with SG only (OR: 2.99, 95% CI: 1.47–6.12) and MCR using 3IR (OR: 3.13, 95% CI: 1.57–6.25) after adjustment for all confounding factors. MCR using 3IR showed synergistic effects on all fall-related outcomes (Table 2).

By contrast, MCR using SCCs was not associated with experience of falls, recurrent falls, falls with injury or falls with fracture (*p* > 0.05), while SG only was significantly associated with experience of falls (OR: 1.87, 95% CI: 1.01–3.45), recurrent falls (OR: 2.27, 95% CI: 1.04–4.96), and falls with injury (OR: 1.87, 95% CI: 1.02–3.42). In addition, both SG only (OR: 3.72, 95% CI: 1.86–7.43) and MCR using SCCs (OR: 2.04, 95% CI: 1.26–3.30) showed a significant association with FoF. Moreover, SG only (OR: 2.72, 95% CI: 1.24–6.00) and MCR using SCCs (OR: 2.75, 95% CI: 1.37–5.50) were associated with low ABC. However, the impact of SG only on most fall-related outcomes was stronger than that of MCR using SCCs.

## 4. Discussion

In this cohort study with 70–84 year-old community-dwelling older adults, the prevalence of the new MCR using 3IR was 4.9% (105/2133), and that of MCR using SCCs was 6.3% (134/2133), which is consistent with other previous studies [6]. The prevalence of MCR using SCCs in the present study was lower than that of our previous study (8.0%) [16]. Such a gap might be due to the different exclusion criteria of the studied populations, especially additional exclusion of dementia based on MMSE score (< 24). The overlap between the newly developed criteria, impaired 3IR, and the conventional criteria of SCCs was 44.2%, which simply assesses subjects’ memory status, but differs in the method of identifying memory deficits. Our main finding is that MCR using 3IR was associated with all fall-related outcomes, including experience of falls, recurrent falls, falls with injury, falls with fracture, FoF, and low ABC, with synergistic effects of its components. By contrast, MCR using SCCs was not associated with falls, recurrent falls, and falls with injury or fracture, and SG only showed a stronger association with most fall-related outcomes.

The idea of a newly attempted MCR using 3IR was developed from our previous findings that SCCs, widely used to define MCR, had a considerably high prevalence rate (77.7%) in older Korean adults, and that the effect of SCCs on cognitive impairment was not additive to SG [16]. Elderly Koreans tend to report more SCCs [15] as well as depressive symptoms [17,45] compared to Western countries. This tendency may be influenced by cultural factors [46]. Several previous studies had shown that SCCs might be more closely associated with depressive symptoms than actual cognitive function [47,48,49]. Therefore, we intended to compensate for the limitations of SCCs for older Korean adults by establishing another criterion for the cognitive aspects of MCR. 

We adopted the 3IR test of MMSE instead of SCCs for definition of MCR based on the following reasons. First, most previous studies identified SCCs using memory-related items [22]. Therefore, we intended to find another tool to represent the subjects’ memory function. Second, the 3IR test is one of the components of MMSE, which has been widely used and validated in many clinical practices and community settings to screen for dementia [24]. Third, some studies have reported that the 3IR was one of the best discriminators among the subscales of MMSE for screening dementia [50]. Fourth, the 3IR test is simple and easy to conduct, which is in line with the usefulness of MCR in various clinical practice settings [9]. In addition, the 3IR test is also a part of the Mini-Cog test, which has high sensitivity and specificity for detecting AD and related dementia in community settings [51]. The Mini-Cog test may not be greatly affected by cultural differences [29]. In our study, the cut-off score to define impaired 3IR (< 3) was based on the first step of the Mini-Cog test, in which participants with a score less than 3 either required an additional step (score 1–2) or were diagnosed as demented (score 0) [29]. 

In the present study, we examined the associations of MCR using a 3IR test with various fall-related outcomes, and compared the results to those of original MCR using SCCs. Callisaya et al. reported that MCR was associated with increased risk of any falls and multiple falls (≥ 2 falls) based on five Western cohort studies, reporting stronger predictive capability of MCR than its individual components [19]. Similarly, in a study in New Zealand comparing the fall risks of Māori and non-Māori populations, MCR and its components were significantly associated with increased fall risks, with synergistic effects of the components in the non-Māori group. However, these associations were not observed in the Māori group [21]. In a French cohort study, subjects with MCR were at higher risk of having experienced falls, recurrent falls, and post-fall fractures, but not those with each component of MCR [20]. Our findings, in terms of the associations of MCR with fall-related outcomes, are consistent with these three previous studies. In our study, MCR using 3IR showed synergistic effects of individual components of MCR compared to MCR using SCCs. 

By contrast, in the associations of MCR using SCCs and its components with fall-related outcomes, SG only had a stronger association than MCR using SCCs, and SCCs only showed protective associations with some fall-related outcomes. These protective associations of SCCs only with most fall-related outcomes, however, became nonsignificant after adjusting for psychological factors, including depressive symptoms and self-reported health status. This change may have been due to the influence of depression or self-rating questionnaire methods on SCCs. Several studies have reported associations between SCCs and depression or self-perceived health [48,52]. In addition, Carrasco et al. reported that the quantity or quality of memory complaints was an important issue for interpreting results, as the self-perceived state of health or mood may be the only factor influencing SCCs, particularly in subjects with fewer complaints [48], which may just be due to normal aging. Therefore, further studies are suggested to examine the effects of psychological factors to further investigate the concept of MCR.

Overall, the associations between MCR, regardless of its definition, and the fall-related outcomes showed specific features in this study. The pattern of the main results was different between each definition of MCR. MCR using 3IR showed a significant association with both fall history retrospectively collected, and self-evaluation of current fear or balance confidence, and SG only showed a significant association only with self-evaluated outcomes. In contrast to this pattern, MCR using SCCs showed a significant association with the self-rated outcomes, and did not show significance in the retrospective fall history. We suspected that these features might be related to the effects of psychological factors on both SCCs and MCR using SCCs [53].

This study had some limitations. First, the causal relationships between MCR, regardless of definition, and fall-related outcomes could not be determined because of the cross-sectional study design. Second, information on some fall-related outcomes was collected retrospectively, which may cause recall bias. Third, the subjects were relatively healthy because they were ambulatory older adults in the community. In addition, the participants were recruited in a research setting. Therefore, our findings may not be generalizable to other settings. Despite these limitations, our findings are important because we included a large, nationally representative sample of older Korean adults, and took into consideration various robust confounders.

## 5. Conclusions

MCR using 3IR was associated with fall-related outcomes, with synergistic effects of the individual components. However, SG only showed stronger effects on most fall-related outcomes than MCR using SCCs. SCCs was susceptible to psychological factors, such as depressive symptoms.

## Figures and Tables

**Table 1:** Characteristics of the participants according to motoric cognitive risk (MCR) syndrome status using three-item recall (3IR).

Characteristics	Overall	Normal (without Impaired 3IR and Slow Gait)	Impaired 3IR only	Slow Gait only	MCR Using 3IR	*p* Value
	(*n* = 2133)	(*n* = 854)	(*n* = 1081)	(*n* = 93)	(*n* = 105)	
***Sociodemographic factors***						
Age (years)	75.6 ± 3.8	75.4 ± 3.9	75.7 ± 3.8	75.2 ± 3.8	75.8 ± 4.0	0.350
Female sex	1032 (48.4)	468 (54.8) ^*^	476 (44.0) ^*^	48 (51.6)	40 (38.1)	**<0.001**
Education (years)	9.7 ± 4.7	9.6 ± 4.7 ^b^	10.1 ± 4.5 ^d^	7.7 ± 5.3 ^b,d^	9.0 ± 4.6	**<0.001**
Residence						
Urban	652 (30.6)	287 (33.6)	311 (28.8)	25 (26.9)	29 (27.6)	**0.008**
Suburban	976 (45.8)	374 (43.8)	523 (48.4)	34 (36.6)	45 (42.9)	
Rural	505 (23.7)	193 (22.6)	247 (22.8) ^*^	34 (36.6)	31 (29.5)	
Living alone	423 (19.8)	178 (20.8)	193 (17.9)	26 (28.0)	26 (24.8)	**0.034**
Marital status (without partner)	614 (28.8)	259 (30.3)	283 (26.2)	37 (39.8)	35 (33.3)	**0.011**
Basic livelihood security *and/or* medical care aid recipient	149 (7.0)	61 (7.1)	61 (5.6)	16 (17.2) ^*^	11 (10.5)	**<0.001**
***Lifestyle-related factors***						
Current smoker	121 (5.7)	43 (5.0)	65 (6.0)	5 (5.4)	8 (7.6)	0.650
Alcohol consumption(≥2 to 3 times/week)	405 (19.0)	150 (17.6)	222 (20.5)	13 (14.0)	20 (19.0)	0.228
Low physical activity	169 (7.9)	54 (6.3) ^*^	75 (6.9)	20 (21.5) ^*^	20 (19.0) ^*^	**<0.001**
***General health and medical conditions***						
BMI (kg/m^2^)	24.5 ± 3.0	24.6 ± 3.0	24.4 ± 2.9 ^d^	25.1 ± 3.0 ^d^	24.8 ± 3.6	0.061
<18.5	34 (1.6)	10 (1.2)	21 (1.9)	0 (0.0)	3 (2.9) ^*^	0.257
18.5–24.9	1213 (56.9)	479 (56.1)	630 (58.3)	48 (51.6)	56 (53.3)	
≥25	886 (41.5)	395 (42.7)	430 (39.8)	45 (48.4)	46 (43.8)	
Number of drugs taken daily	3.4 ± 2.9	3.3 ± 2.8 ^b,c^	3.3 ± 2.9 ^d,e^	4.7 ± 3.8 ^b,d^	4.2 ± 3.0 ^c,e^	**<0.001**
Number of diseases	1.7 ± 1.2	1.7 ± 1.2 ^c^	1.6 ± 1.2 ^e^	1.9 ± 1.2	2.0 ± 1.3 ^c,e^	**0.004**
Hypertension	1211 (56.8)	476 (55.7)	611 (56.5)	55 (59.1)	69 (65.7)	0.258
Diabetes	458 (21.5)	160 (18.7)	236 (21.8)	23 (24.7)	39 (37.1) ^*^	**<0.001**
Dyslipidemia	718 (33.7)	304 (35.6)	353 (32.7)	27 (29.0)	34 (32.4)	0.408
Urinary incontinence	65 (3.0)	29 (3.4)	29 (2.7)	5 (5.4)	2 (1.9)	0.390
Visual impairment	39 (1.8)	14 (1.6)	18 (1.7)	4 (4.3)	3 (2.9)	0.230
Hearing impairment	325 (15.2)	126 (14.8)	166 (15.4)	15 (16.1)	18 (17.1)	0.915
Poor nutritional status (MNA screening score ≤ 11)	144 (6.8)	56 (6.6)	69 (6.4)	11 (11.8)	8 (7.6)	0.239
IADL disability	246 (11.5)	109 (12.8)	107 (9.9) ^*^	19 (20.4) ^*^	11 (10.5) ^*^	**0.010**
***Psychological factors***						
General cognitive function (MMSE score)	27.0 ± 1.7	27.8 ± 1.7 ^a,c^	26.4 ± 1.4 ^a,d^	27.6 ± 2.0 ^d,f^	26.0 ± 1.4 ^c,f^	**<0.001**
Fair/poor self-perceived health	533 (25.0)	201 (23.5)	244 (22.6)	45 (48.4) ^*^	43 (41.0) ^*^	**<0.001**
Depressive symptoms (GDS score ≥ 6)	383 (18.0)	141 (16.5)	179 (16.6)	31 (33.3) ^*^	32 (30.5) ^*^	**<0.001**
Quality of life (EQ-5D score)	0.899 ± 0.117	0.903 ± 0.113 ^b,c^	0.909 ± 0.111 ^d,e^	0.824 ± 0.155 ^b,d^	0.830 ± 0.132 ^c,e^	**0.007**
***Physical functions***						
Handgrip strength (kg)	27.4 ± 7.5	27.0 ± 7.4 ^a^	27.9 ± 7.6 ^a^	25.9 ± 7.6	26.7 ± 6.9	**<0.001**
Usual walking speed (m/s)	1.14 ± 0.24	1.19 ± 0.21 ^b,c^	1.17 ± 0.22 ^d,e^	0.78 ± 0.13 ^b,d^	0.78 ± 0.13 ^c,e^	**<0.001**
Timed get up and go test (s) ^†^	10.0 ± 2.2	9.6 ± 2.0 ^b,c^	9.7 ± 1.8 ^d,e^	12.5 ± 3.4 ^b,d^	12.5 ± 3.1 ^c,e^	**<0.001**
SPPB score ^†^	11.1 ± 1.3	11.2 ± 1.1 ^b,c^	11.2 ± 1.1 ^d,e^	10.1 ± 2.0 ^b,d^	9.9 ± 1.9 ^c,e^	**<0.001**
***MCR syndrome Using three-item recall test***						
Impaired three-item recall	1186 (55.6)	0 (0)	1081 (100)	0 (0)	105 (100)	**<0.001**
Slow gait	198 (9.3)	0 (0)	0 (0)	93 (100)	105 (100)	**<0.001**

*Notes:* Values are mean ± SD, *n* (%). *Abbreviations:* SD = Standard deviation; BMI = Body Mass Index; IADL = Instrumental Activities of Daily Living; MNA = Mini Nutritional Assessment; GDS = Geriatric Depression Scale; SPPB = Short Physical Performance Battery; MCR = Motoric Cognitive Risk; MMSE = Mini Mental State Examination. ^a^ Comparison between normal group and impaired 3IR only group; ^b^ comparison between normal group and slow gait only group; ^c^ comparison between normal group and MCR using 3IR group; ^d^ comparison between impaired 3IR only group and slow gait only group; ^e^ comparison between impaired 3IR only group and MCR using 3IR group; ^f^ comparison between slow gait only group and MCR using 3IR group. with Bonferroni post hoc test or Welch’s ANOVA with Games-Howell post hoc test for continuous variables. *P* < 0.05 indicated in bold. ^*^ Significance with *P* < 0.05 divided by the number of rows × columns, with post hoc test of chi-square tests. ^§^ Sample size. ^†^ Some missing data. ^‡^ *P*-values were calculated using the Chi-square test or Fisher’s exact test for categorical variables and one-way ANOVA.

**Table 2:** Associations between motoric cognitive risk (MCR) syndrome using three-item recall (3IR) and the fall-related outcomes (*n* = 2133).

Dependent Variables	Odds Ratio (95% Confidence Interval) (*p*-Value)						
	Normal (without Impaired 3IR and Slow Gait)	Impaired 3IR only	*p*	Slow Gait only	*p*	MCR Using 3IR	*p*
***Experience of falls in the past 1 year***							
Model 1	Ref.	0.971 (0.754, 1.251)	0.820	1.406 (0.823, 2.402)	0.212	2.157 (1.282, 2.255)	**0.002**
Model 2		0.969 (0.753, 1.248)	0.809	1.349 (0.784, 2.320)	0.279	2.098 (1.288, 3.416)	**0.003**
Model 3		0.972 (0.753, 1.254)	0.827	1.257 (0.725, 2.179)	0.414	2.080 (1.271, 3.404)	**0.004**
Model 4		0.959 (0.742, 1.239)	0.748	1.166 (0.667, 2.038)	0.590	1.915 (1.160, 3.160)	**0.011**
***Recurrent falls (≥ twice)***							
Model 1	Ref.	0.789 (0.523, 1.189)	0.257	1.881 (0.921, 3.840)	0.083	2.745 (1.446, 5.213)	**0.002**
Model 2		0.785 (0.521, 1.184)	0.248	1.686 (0.811, 3.505)	0.162	2.503 (1.302, 4.811)	**0.006**
Model 3		0.809 (0.535, 1.223)	0.315	1.563 (0.745, 3.280)	0.237	2.581 (1.329, 5.012)	**0.005**
Model 4		0.778 (0.513, 1.180)	0.237	1.361 (0.642, 2.889)	0.422	2.194 (1.115, 4.318)	**0.023**
***Falls with injury***							
Model 1	Ref.	0.980 (0.766, 1.252)	0.869	1.647 (0.992, 2.735)	0.054	2.207 (1.380, 3.530)	**0.001**
Model 2		0.977 (0.764, 1.250)	0.856	1.585 (0.949, 2.648)	0.079	2.151 (1.340, 3.454)	**0.002**
Model 3		0.980 (0.765, 1.255)	0.871	1.493 (0.888, 2.511)	0.131	2.141 (1.328, 3.452)	**0.002**
Model 4		0.967 (0.754, 1.240)	0.790	1.392 (0.821, 2.360)	0.219	1.982 (1.220, 3.220)	**0.006**
***Falls with fracture***							
Model 1	Ref.	0.660 (0.373, 1.165)	0.660	2.442 (0.996, 5.987)	0.051	3.133 (1.404, 6.988)	**0.005**
Model 2		0.659 (0.373, 1.164)	0.151	1.967 (0.780, 4.961)	0.152	2.764 (1.224, 6.237)	**0.014**
Model 3		0.662 (0.372, 1.180)	0.162	1.727 (0.671, 4.444)	0.257	2.722 (1.185, 6.251)	**0.018**
Model 4		0.648 (0.363, 1.157)	0.143	1.593 (0.608, 4.171)	0.343	2.508 (1.086, 5.791)	**0.031**
***Fear of falling***							
Model 1	Ref.	0.969 (0.769, 1.221)	0.789	3.090 (1.901, 5.023)	**<0.001**	3.851 (2.409, 6.157)	**<0.001**
Model 2		0.967 (0.767, 1.218)	0.776	2.885 (1.764, 4.720)	**<0.001**	3.664 (2.283, 5.878)	**<0.001**
Model 3		0.981 (0.776, 1.241)	0.875	2.604 (1.575, 4.305)	**<0.001**	3.407 (2.111, 5.497)	**<0.001**
Model 4		0.954 (0.751, 1.212)	0.700	2.218 (1.314, 3.746)	**0.003**	3.000 (1.830, 4.917)	**<0.001**
***Low activities-specific balance confidence***							
Model 1	Ref.	1.037 (0.713, 1.508)	0.849	5.403 (2.960, 9.863)	**<0.001**	5.269 (2.881, 9.639)	**<0.001**
Model 2		1.010 (0.692, 1.474)	0.957	4.358 (2.335, 8.135)	**<0.001**	4.609 (2.477, 8.576)	**<0.001**
Model 3		1.087 (0.733, 1.613)	0.678	4.094 (2.087, 8.032)	**<0.001**	4.320 (2.268, 8.230)	**<0.001**
Model 4		0.978 (0.648, 1.478)	0.917	2.994 (1.467, 6.108)	**0.003**	3.134 (1.571, 6.253)	**0.001**

*Notes:* Model 1: Adjusted for recruited center and sociodemographic factors; age, sex, low education level, residence (urban/suburban/rural), living alone, without partner, receiving basic livelihood security and/or medical care aid. Model 2: Further adjustment on Model 1 for lifestyle-related factors; current smoker, alcohol consumption, low physical activity. Model 3: Further adjustment on Model 2 for general health and medical conditions; body mass index (underweight/normal/obese), number of drugs taken daily, number of diseases (self-reported doctor diagnosis of hypertension, diabetes mellitus, dyslipidemia, myocardial infarction, congestive heart failure, angina pectoris, cerebrovascular disease, peripheral vascular disease, osteoarthritis, rheumatoid arthritis, osteoporosis, asthma, and chronic obstructive pulmonary disease), urinary incontinence, visual impairment, hearing impairment, poor nutritional status, number of difficulties in instrumental activities of daily living (IADL). Model 4: Further adjustment on Model 4 for psychological factors; depressive symptoms, fair/poor self-reported health status. *p* < 0.05 indicated in bold.

**Table 3:** Associations between motoric cognitive risk (MCR) syndrome using subjective cognitive complaints (SCCs) and the fall-related outcomes (*n* = 2133).

Dependent Variables	Odds Ratio (95% Confidence Interval) (*p*-Value)						
	Normal (without SCCs and Slow Gait)	SCCs only	*p*	Slow Gait only	*p*	MCR Using SCCs	*p*
***Experience of falls in the past 1 year***							
Model 1	Ref.	0.705 (0.519, 0.959)	**0.026**	2.047 (1.133, 3.699)	**0.018**	1.080 (0.647, 1.801)	0.769
Model 2		0.711 (0.523, 0.967)	**0.029**	1.989 (1.095, 3.612)	**0.024**	1.061 (0.634, 1.774)	0.823
Model 3		0.770 (0.562, 1.055)	0.103	1.955 (1.066, 3.586)	**0.030**	1.123 (0.667, 1.888)	0.663
Model 4		0.902 (0.650, 1.253)	0.540	1.865 (1.008, 3.452)	**0.047**	1.254 (0.740, 2.126)	0.400
***Recurrent falls (≥ twice)***							
Model 1	Ref.	0.531 (0.336, 0.840)	**0.007**	2.631 (1.255, 5.517)	**0.010**	1.165 (0.574, 2.367)	0.672
Model 2		0.550 (0.346, 0.872)	**0.011**	2.430 (1.145, 5.156)	**0.021**	1.106 (0.539, 2.271)	0.784
Model 3		0.609 (0.378, 0.979)	**0.041**	2.411(1.113, 5.220)	**0.026**	1.214 (0.586, 2.513)	0.601
Model 4		0.790 (0.479, 1.304)	0.356	2.269 (1.038, 4.958)	**0.040**	1.410 (0.672, 2.960)	0.364
***Falls with injury***							
Model 1	Ref.	0.709 (0.526, 0.955)	**0.024**	2.021 (1.127, 3.622)	**0.018**	1.258 (0.776, 2.040)	0.352
Model 2		0.716 (0.531, 0.965)	**0.028**	1.961 (1.089, 3.533)	**0.025**	1.241 (0.763, 2.047)	0.384
Model 3		0.766 (0.565, 1.040)	0.088	1.950 (1.073, 3.541)	**0.028**	1.299 (0.794, 2.123)	0.297
Model 4		0.897 (0.652, 1.235)	0.505	1.864 (1.018, 3.416)	**0.044**	1.454 (0.882, 2.396)	0.142
***Falls with fracture***							
Model 1	Ref.	0.969 (0.473, 1.983)	0.931	7.533 (2.893, 19.614)	**<0.001**	1.682 (0.580, 4.874)	0.338
Model 2		0.998 (0.485, 2.051)	0.995	6.678 (2.528, 17.638)	**<0.001**	1.449 (0.493, 4.254)	0.500
Model 3		1.193 (0.561, 2.534)	0.647	8.001 (2.898, 22.094)	**<0.001**	1.479 (0.491, 4.458)	0.487
Model 4		1.491 (0.679, 3.273)	0.319	7.738 (2.766, 21.651)	**<0.001**	1.763 (0.575, 5.410)	0.321
***Fear of falling***							
Model 1	Ref.	0.566 (0.428, 0.747)	**<0.001**	4.245 (2.223, 8.103)	**<0.001**	1.712 (1.088, 2.696)	**0.020**
Model 2		0.569 (0.431, 0.752)	**<0.001**	4.047 (2.107, 7.773)	**<0.001**	1.632 (1.033, 2.580)	**0.036**
Model 3		0.644 (0.484, 0.857)	**0.003**	3.756 (1.945, 7.252)	**<0.001**	1.701 (1.068, 2.709)	**0.025**
Model 4		0.874 (0.644, 1.185)	0.385	3.719(1.861, 7.432)	**<0.001**	2.040 (1.260, 3.301)	**0.004**
***Low activities-specific balance confidence***							
Model 1	Ref.	0.414 (0.276, 0.621)	**<0.001**	3.951 (2.019, 7.733)	**<0.001**	2.198 (1.211, 3.990)	**0.010**
Model 2		0.434 (0.288, 0.654)	**<0.001**	3.449 (1.727, 6.890)	**<0.001**	1.952 (1.056, 3.610)	**0.033**
Model 3		0.561 (0.362, 0.870)	**0.010**	3.520 (1.683, 7.362)	**0.001**	2.215 (1.153, 4.254)	**0.017**
Model 4		0.829 (0.517, 1.331)	0.437	2.722 (1.235, 6.001)	**0.013**	2.748 (1.374, 5.495)	**0.004**

*Notes:* Model 1: Adjusted for recruited center and sociodemographic factors; age, sex, low education level, residence (urban/suburban/rural), living alone, without partner, receiving basic livelihood security and/or medical care aid. Model 2: Further adjustment on Model 1 for lifestyle-related factors; current smoker, alcohol consumption, low physical activity. Model 3: Further adjustment on Model 2 for general health and medical conditions; body mass index (underweight/normal/obese), number of drugs taken daily, number of diseases (self-reported doctor diagnosis of hypertension, diabetes mellitus, dyslipidemia, myocardial infarction, congestive heart failure, angina pectoris, cerebrovascular disease, peripheral vascular disease, osteoarthritis, rheumatoid arthritis, osteoporosis, asthma, and chronic obstructive pulmonary disease), urinary incontinence, visual impairment, hearing impairment, poor nutritional status, number of difficulties in instrumental activities of daily living (IADL). Model 4: Further adjustment on Model 4 for psychological factors; depressive symptoms, fair/poor self-reported health status. *P* < 0.05 indicated in bold.

## Supplementary Materials

The following are available online at https://www.mdpi.com/1660-4601/17/10/3364/s1, Table S1: Characteristics of participants according to MCR status using subjective cognitive complaints (SCCs).
