# Supplementary material for: Motoric Cognitive Risk Syndrome Using Three-Item Recall Test and Its Associations with Fall-Related Outcomes: The Korean Frailty and Aging Cohort Study
Source: Int J Environ Res Public Health. 2020 May 12;17(10):3364. doi: 10.3390/ijerph17103364 (PMC7277733; doi:10.3390/ijerph17103364)
Supplement: Supplementary file 1 [file ijerph-17-03364-s001.zip › ijerph-793168-supplementary.docx]

**Supplementary materials**

**Table S1**. Characteristics of participants according to MCR status using subjective cognitive complaints (SCCs).

| **Characteristics** | **Overall** | **Normal**  **(without SCCs**  **and slow gait)** | **SCCs**  **only** | **Slow gait**  **only** | **MCR using SCCs** | ***P* Value^‡^** |
| --- | --- | --- | --- | --- | --- | --- |
|  | **(*n* = 2133)** | **(*n* = 341)** | **(*n* = 1594)** | **(*n* = 64)** | **(*n* = 134)** |  |
| ***Sociodemographic factors*** |  |  |  |  |  |  |
| Age (years) | 75.6 $\pm$3.8 | 75.4 $\pm$3.9 | 75.7 $\pm$3.8 | 75.2 $\pm$3.8 | 75.8 $\pm$4.0 | 0.350 |
| Female sex | 1032 (48.4) | 175 (51.3) | 769 (48.2) | 36 (56.3) | 52 (38.8) | 0.053 |
| Education (years) | 9.7 $\pm$4.7 | 8.6 $\pm$4.5 ^a,b^ | 10.2 $\pm$4.6 ^a,d^ | 6.7 $\pm$5.1 ^b,d,f^ | 9.1 $\pm$4.7 ^f^ | **<0.001** |
| Residence |  |  |  |  |  |  |
| Urban | 652 (30.6) | 92 (27.0) | 506 (31.7) | 11 (17.2) | 43 (32.1) | **0.002** |
| Suburban | 976 (45.8) | 158 (46.3) | 739 (46.4) | 27 (42.2) | 52 (38.8) |  |
| Rural | 505 (23.7) | 91 (26.7) | 349 (21.9) | 26 (40.6) | 39 (29.1) |  |
| Living alone | 423 (19.8) | 81 (23.8) | 290 (18.2) | 20 (31.3) | 32 (23.9) | **0.006** |
| Marital status (without partner) | 614 (28.8) | 107 (31.4) | 435 (27.3) | 28 (43.8) | 44 (32.8) | **0.012** |
| Basic livelihood security  *and/or* medical care aid recipient | 149 (7.0) | 24 (7.0) | 98 (6.1) | 11 (17.2) | 16 (11.9) | **0.001** |
| ***Lifestyle-related factors*** |  |  |  |  |  |  |
| Current smoker | 121 (5.7) | 19 (5.6) | 89 (5.6) | 5 (7.8) | 8 (6.0) | 0.896 |
| Alcohol consumption  (≥ 2 to 3 times/week) | 405 (19.0) | 68 (19.9) | 304 (19.1) | 10 (15.6) | 23 (17.2) | 0.809 |
| Low physical activity | 169 (7.9) | 29 (8.5) | 100 (6.3) | 18 (28.1) | 22 (16.4) | **<0.001** |
| ***General health and medical conditions*** |  |  |  |  |  |  |
| BMI (kg/m^2^) | 24.5 $\pm$3.0 | 24.4 $\pm$2.8 | 24.5 $\pm$2.9 | 24.9 $\pm$3.4 | 25.0 $\pm$3.3 | 0.180 |
| < 18.5 | 34 (1.6) | 4 (1.2) | 27 (1.7) | 1 (1.6) | 2 (1.5) | 0.788 |
| 18.5–24.9 | 1213 (56.9) | 204 (59.8) | 905 (56.8) | 33 (51.6) | 71 (53.0) |  |
| ≥ 25 | 886 (41.5) | 133 (39.0) | 662 (41.5) | 30 (46.9) | 61 (45.5) |  |
| Number of drugs taken daily | 3.4 $\pm$2.9 | 4.1 $\pm$3.4 ^a^ | 3.2 $\pm$2.7 ^a,e^ | 4.3 $\pm$3.4 | 4.6 $\pm$3.3 ^e^ | **<0.001** |
| Number of diseases | 1.7 $\pm$1.2 | 1.9 $\pm$1.3 ^a^ | 1.6 $\pm$1.2 ^a,d,e^ | 2.1 $\pm$1.2 ^d^ | 1.9 $\pm$1.3 ^e^ | **<0.001** |
| Hypertension | 1211 (56.8) | 190 (55.7) | 897 (56.3) | 43 (67.2) | 81 (60.4) | 0.274 |
| Diabetes | 458 (21.5) | 87 (25.5) | 309 (19.4) | 18 (28.1) | 44 (32.8) | **<0.001** |
| Dyslipidemia | 718 (33.7) | 116 (34.0) | 541 (33.9) | 18 (28.1) | 43 (32.1) | 0.777 |
| Urinary incontinence | 65 (3.0) | 13 (3.8) | 45 (2.8) | 2 (3.1) | 5 (3.7) | 0.761 |
| Visual impairment | 39 (1.8) | 6 (1.8) | 26 (1.6) | 1 (1.6) | 6 (4.5) | 0.126 |
| Hearing impairment | 325 (15.2) | 53 (15.5) | 239 (15.0) | 13 (20.3) | 20 (14.9) | 0.709 |
| Poor nutritional status  (MNA screening score ≤ 11) | 144 (6.8) | 35 (10.3) | 90 (5.6) | 11 (17.2) | 8 (6.0) | **<0.001** |
| IADL disability | 246 (11.5) | 52 (15.2) | 164 (10.3) | 17 (26.6) | 13 (9.7) | **<0.001** |
| ***Psychological factors*** |  |  |  |  |  |  |
| General cognitive function (MMSE score) | 27.0 $\pm$1.7 | 26.6 $\pm$1.7 ^a^ | 27.1 $\pm$1.7 ^a,d^ | 26.3 $\pm$1.9 ^d^ | 27.0 $\pm$1.8 | **<0.001** |
| Fair/poor self-perceived health | 533 (25.0) | 139 (40.8) | 306 (19.2) | 39 (60.9) | 49 (36.6) | **<0.001** |
| Depressive symptoms (GDS score ≥ 6) | 383 (18.0) | 132 (38.7) | 188 (11.8) | 35 (54.7) | 28 (20.9) | **<0.001** |
| Quality of life (EQ-5D score) | .899 $\pm$.117 | .871 $\pm$.130 ^a,b^ | .914 $\pm$.106 ^a,d,e^ | .771 $\pm$.163 ^b,d,f^ | .854 $\pm$.124 ^e,f^ | **<0.001** |
| ***Physical functions*** |  |  |  |  |  |  |
| Handgrip strength (kg) | 27.4 $\pm$7.5 | 26.6 $\pm$7.7 | 27.7 $\pm$7.5 ^d^ | 25.1 $\pm$7.6 ^d^ | 26.9 $\pm$7.0 | **<0.001** |
| Usual walking speed (m/s) | 1.14 $\pm$0.24 | 1.15 $\pm$0.22 ^a,b^ | 1.19 $\pm$0.21 ^a,d,e^ | 0.76 $\pm$0.12^b,d^ | 0.79 $\pm$0.13 ^e^ | **<0.001** |
| Timed get up and go test (s)^†^ | 10.0 $\pm$2.2 | 10.2 $\pm$2.1 ^a,b,c^ | 9.6 $\pm$1.8 ^a,d,e^ | 13.2 $\pm$3.9 ^b,d^ | 12.2 $\pm$2.8 ^c,e^ | **<0.001** |
| SPPB score^†^ | 11.1 $\pm$1.3 | 11.0 $\pm$1.2 ^a,b,c^ | 11.3 $\pm$1.1 ^a,d,e^ | 9.6 $\pm$1.8 ^b,d^ | 10.2 $\pm$2.0 ^c,e^ | **<0.001** |
| ***Fall-related factors*** |  |  |  |  |  |  |
| Fall experience in the past one year | 355 (16.6) | 69 (20.2) | 236 (14.8) | 23 (35.9) | 27 (20.1) | **<0.001** |
| Number of falls | 0.3 $\pm$1.1 | 0.4 $\pm$1.0 | 0.3 $\pm$1.0 ^d^ | 0.7 $\pm$1.2 ^d^ | 0.6 $\pm$2.0 | **0.005** |
| Recurrent falls ($\geq$ twice) | 128 (6.0) | 29 (8.5) | 73 (4.6) | 13 (20.3) | 13 (9.7) | **<0.001** |
| Falls with injury | 385 (18.0) | 74 (21.7) | 254 (15.9) | 24 (37.5) | 33 (24.6) | **<0.001** |
| Falls with fracture | 69 (3.2) | 10 (2.9) | 42 (2.6) | 11 (17.2) | 6 (4.5) | **<0.001** |
| Fear of falling | 581 (27.2) | 122 (35.8) | 355 (22.3) | 46 (71.9) | 58 (43.3) | **<0.001** |
| Activities-specific balance confidence score | 83.1 $\pm$16.5 | 76.8 $\pm$18.1^a,b^ | 85.9 $\pm$14.2 ^a,d,e^ | 62.4 $\pm$22.8 ^b,d,f^ | 75.8 $\pm$20.3 ^e,f^ | **<0.001** |
| ***MCR syndrome Using SCCs*** |  |  |  |  |  |  |
| Subjective cognitive complaints | 1728 (81.0) | 0 (0) | 1594 (100) | 0 (0) | 134 (100) | **<0.001** |
| Slow gait | 198 (9.3) | 0 (0) | 0 (100) | 64 (1000) | 134 (100) | **<0.001** |
| *Notes*: Values are mean ± SD, n (%).  *Abbreviations*: SD = Standard deviation; BMI = Body Mass Index; IADL = Instrumental Activities of Daily Living; MNA = Mini Nutritional Assessment; GDS = Geriatric Depression Scale; SPPB = Short Physical Performance Battery; MCR = Motoric Cognitive Risk; MMSE = Mini Mental State Examination.  ^a^ Comparison between normal group and SCCs only group; ^b^ comparison between normal group and slow gait only group; ^c^ comparison between normal group and MCR using SCCs group; ^d^ comparison between SCCs only group and slow gait only group; ^e^ comparison between SCCs only group and MCR using SCCs group; ^f^ comparison between slow gait only group and MCR using SCCs group.  *Sample size.  †Some missing data.  ‡P values were calculated using the Chi-square test or Fisher’s exact test for categorical variables and one-way ANOVA with Bonferroni post hoc test or Welch’s ANOVA with Games-Howell post hoc test for continuous variables. *P* < 0.05 indicated in bold. | | | | | | |
